# Detangling the Crosstalk Between *Ascaris, Trichuris* and Gut Microbiota: What´s Next?

**DOI:** 10.3389/fcimb.2022.852900

**Published:** 2022-05-25

**Authors:** Sergio Castañeda, Alberto Paniz-Mondolfi, Juan David Ramírez

**Affiliations:** ^1^ Centro de Investigaciones en Microbiología y Biotecnología-UR (CIMBIUR), Facultad de Ciencias Naturales, Universidad del Rosario, Bogotá, Colombia; ^2^ Molecular Microbiology Laboratory, Department of Pathology, Molecular and Cell-Based Medicine, Icahn School of Medicine at Mount Sinai, New York, NY, United States

**Keywords:** microbiota, helminths, *Trichuris*, *Ascaris*, host-parasite interactions

## Abstract

Helminth infections remain a global public health issue, particularly in low- and middle-income countries, where roundworms from the*Trichuris* and *Ascaris* genera are most prevalent. These geohelminths not only impact human health but most importantly also affect animal well-being, in particular the swine industry. Host-helminth parasite interactions are complex and at the same time essential to understand the biology, dynamics and pathophysiology of these infections. Within these interactions, the immunomodulatory capacity of these helminths in the host has been extensively studied. Moreover, in recent years a growing interest on how helminths interact with the intestinal microbiota of the host has sparked, highlighting how this relationship plays an essential role in the establishment of initial infection, survival and persistence of the parasite, as well as in the development of chronic infections. Identifying the changes generated by these helminths on the composition and structure of the host intestinal microbiota constitutes a field of great scientific interest, since this can provide essential and actionable information for designing effective control and therapeutic strategies. Helminths like *Trichuris* and *Ascaris* are a focus of special importance due to their high prevalence, higher reinfection rates, resistance to anthelmintic therapy and unavailability of vaccines. Therefore, characterizing interactions between these helminths and the host intestinal microbiota represents an important approach to better understand the nature of this dynamic interface and explore novel therapeutic alternatives based on management of host microbiota. Given the extraordinary impact this may have from a biological, clinical, and epidemiological public health standpoint, this review aims to provide a comprehensive overview of current knowledge and future perspectives examining the parasite-microbiota interplay and its impact on host immunity.

## Introduction

Helminth infections represent a major global public health burden, primarily in developing countries, where lack of basic water, hygiene and sanitation conditions are widespread (Harhay et al., 2010; Hotez et al., 2014; Ojha et al., 2014; Silver et al., 2018) due to stressed socioeconomic conditions. Although many helminth infections are asymptomatic, they can display a variety of clinical presentations, particularly in children, where they are usually associated with growth retardation, malnutrition, and diarrheal disease, among other conditions. Helminth infections have also been linked with a decreased occurrence of autoimmune diseases presumably based on their immunomodulatory capacity (Cooper and Figuieredo, 2013; Girgis et al., 2013; Giacomin et al., 2015a; Maizels et al., 2018; White et al., 2020). It has been largely hypothesized that individuals with increased exposure to helminths during their first years of life are prone to immunomodulatory effects and immunological tolerance induced by parasites, thus decreasing their chances to develop allergic and autoimmune diseases, a phenomenon also known as the “hygiene hypothesis”, later called the “old friends hypothesis” (Altmann, 2009; Chammartin et al., 2013; Stiemsma et al., 2015; Gazzinelli-Guimaraes and Nutman, 2018). It has also been proposed that for certain specific infections such as tuberculosis, helminths may play a relevant role in susceptibility to infection and disease progression, which is correlated with the immunomodulatory properties attributed to helminths (Karo-Atar et al., 2021). Among the most prevalent soil-transmitted helminths worldwide are the genera *Trichuris* and *Ascaris*, which not only affect human health but also have a significant impact on animals and livestock production (Hotez et al., 2008; Harhay et al., 2010; Pullan et al., 2014; Schüle et al., 2014; Else et al., 2020; Gordon et al., 2020).

Studies on the interaction between *Ascaris* and *Trichuris* helminths and host has allowed to determine that in addition to their recognized immunomodulatory capacity, these helminths reveal complex interfaces with the hosts gastrointestinal microbiota, a key factor that may play a fundamental role in parasite survival and persistence (Lee et al., 2014; Zaiss et al., 2015; Zaiss and Harris, 2016; Jenkins et al., 2017; Brosschot and Reynolds, 2018). This host-parasite relationship represents an emerging field of great scientific interest in helminthology and has therefore been subject of intensive scrutiny in recent years. Further evidence showing how the presence of these helminths can relate with changes in the composition and structure of host intestinal microbiota suggests that such interaction may play a determining part in the biology and pathophysiology of the parasite (Lee et al., 2014; Zaiss and Harris, 2016; Ajibola et al., 2019; Cortés et al., 2020; Schachter et al., 2020; Rosa et al., 2021; von Huth et al., 2021). However, contrasting evidence obtained mainly from descriptive studies examining human populations reveals that helminth infections do not exert a significant impact on their interaction with the host microbiota, highlighting that the use of different methodological approaches may derive in heterogeneous results (Cooper et al., 2013; Mejia et al., 2020). Nevertheless, to control potential confusion variables, both for *Trichuris* and *Ascaris*, murine and porcine animal models have been developed and implemented to model the infection process in humans and thus study parasite-host-microbiota interactions, evaluating the changes caused by these helminths in the microbiota in relation to its composition and structure and in order to examine helminth infections and their dynamic interaction with the intestinal microbiota. However, it is also important to highlight the limitations of animal studies, since the similarities and differences in terms of genetics, physiology, diet, etc. must be considered, which is why the use of humanized murine models has been chosen to replicate infection scenarios in humans more efficiently (Lewis et al., 2006; Klementowicz et al., 2012; Nguyen et al., 2015; Williams et al., 2017; Wang et al., 2019; Park and Im, 2020; Yousefi et al., 2021). Likewise, the development and advancement of new sequencing platforms and techniques has promoted a great increase in the availability of data from different approaches. This, together with the continuous innovation of bioinformatics tools, has established novel frameworks that offer interesting strategies in the field of parasite-host-microbiota interactions (Wooley et al., 2010; Simon and Daniel, 2011; Vollmers et al., 2017; Wajid et al., 2022).

Considering the relevance of this field of research, this review will examine in detail the interaction between the intestinal microbiota and helminth parasites, *Trichuris* and *Ascaris*, as well as how these interactions can be potentially influence infection, dissemination, persistence, and survival in the human host.

## Relevance of *Trichuris* and *Ascaris* Helminths

Trichuriasis is caused by the human whipworm *Trichuris trichiura*, which typically exhibits high parasite burdens that usually translate clinically into a severe constellation of symptoms including diarrhea, abdominal pain, malnutrition, stunted growth and developmental arrest of infected children (Chammartin et al., 2013; World Health Organization Soil-transmitted helminth infections, 2021). The *Trichuris* (Nematoda: Trichuridae) parasite is one of the most highly prevalent intestinal helminths worldwide, mainly in Africa and South America, and is estimated to infect about 500 million people globally (Hotez et al., 2014; Pullan et al., 2014; World Health Organization Soil-transmitted helminth infections, 2021). *Trichuris* was predicted to be responsible for the loss of 0.64 million disability-adjusted life years between 1990 and 2010, ranking tenth amongst all neglected tropical diseases (Hotez et al., 2014).

Similarly, infections to *Ascaris* (Nematoda: Ascarididae) species are also amongst the most cosmopolitan helminth infections worldwide and also included within the Neglected Tropical Diseases roster (Saboyá et al., 2013; Pullan et al., 2014; World Health Organization Soil-transmitted helminth infections, 2021). The overall prevalence of helminth infections exceeds 10% in most developing countries with a large percentage of these attributable to *Ascaris lumbricoides* infections, mostly in endemic regions of Africa and South America (Hotez et al., 2014; Pullan et al., 2014; World Health Organization Soil-transmitted helminth infections, 2021). Ascaridiasis has also been associated with malnutrition and developmental delay due to persistent infections, and the disease burden is estimated at around 60,000 deaths per year, mostly in children (Harhay et al., 2010; Hotez et al., 2014; Pullan et al., 2014; Jourdan et al., 2018; Corvino et al., 2021) .

A challenging aspect in the management of this parasitic infections is its well-known resistance to routine anti-helminth therapy and thus, their proneness for reinfections, which has stirred the search for new therapeutic alternatives and the development of potential vaccines. This acquires more relevance in endemic regions, where recurrent infections with low parasite loads may lead to chronic and persistent infections (Harhay et al., 2010; Ramanan et al., 2016; Midha et al., 2017; Papaiakovou et al., 2021; Whitman et al., 2021). Factors such as the highly resistant nature of the eggs in moist soil, long standing patency, high rate of reinfections despite effective anti-helminth therapy and the unavailability of vaccines, have all been associated with a high dispersal of *Ascaris* and *Trichuris*, not only in human hosts but also in other vertebrate hosts (Schüle et al., 2014; Easton et al., 2019; Cools et al., 2021). In addition to the human parasite *A. lumbricoides*, ascariasis infection of swine by the highly prevalent species *Ascaris suum* is also known for its pathogenic effects in domestic pigs and its impact in livestock productivity (Dold and Holland, 2011; Velasco et al., 2011; Cooper and Figuieredo, 2013; Miller et al., 2015; Katakam et al., 2016; Williams et al., 2017; Midha et al., 2018; Hansen et al., 2019; Arora et al., 2020; Easton et al., 2020).

## Host-Helminth-Gut Microbiota Interactions

In an attempt to better understand the host-helminth-gut microbiota relationships, most studies have focused on deciphering interactions between helminths and the host immune system given the important immunomodulatory and immunoregulatory effects exerted by these parasites and how this dynamic regulation influences infectiveness, dissemination and persistence in the infected host (McSorley and Maizels, 2012; Giacomin et al., 2015a; Briggs et al., 2016; Maizels et al., 2018; Yasuda and Nakanishi, 2018; White et al., 2020). Also, recent studies have explored the functional consequences of helminth-induced changes in microbiota and its impact in parasite biology and infection dynamics. Interestingly, changes in the relative abundance of certain bacterial taxa as well as the diversity of the intestinal microbiota in presence of helminths have shown to display profound effects on the parasite life cycle by inducing metabolic changes that could promote infectiveness and dissemination, while modulating the host immune response leading to persistence (Berrilli et al., 2012; Li et al., 2012; Cooper et al., 2013; Cantacessi et al., 2014; Glendinning et al., 2014; Lee et al., 2014; Kay et al., 2015; Zaiss et al., 2015; Giacomin et al., 2015b; Midha et al., 2017; Midha et al., 2018; Rosa et al., 2018; Su et al., 2018; Ajibola et al., 2019; Easton et al., 2019; Wang et al., 2019; Mejia et al., 2020; Rosa et al., 2021). Understanding the mechanisms by which helminth parasites influence the complex microbiota ecosystem of the host and how these changes functionally impact the host intestinal microbiota is critically important, as it will pave the way in search of new control strategies and therapeutic approaches.

Studies addressing the relationship of parasites with host microbiota have shown variable results, particularly in human-based descriptive studies (Cooper et al., 2013; Cantacessi et al., 2014; Jenkins et al., 2017; Cortés et al., 2020). This in part, because many methodological and inherent aspects within study groups are prone to introducing biases, thus generating diverse results and conclusions, such as those related to the study population, impact of diet, environmental factors as well as genetic background (Cooper et al., 2013; Rosa et al., 2018; Rinninella et al., 2019). A critical evaluation of current methodologies deserves a separate and dedicated review. However, in light of the above, the use of animal models, essentially pigs and mice, despite certain limitations that must be carefully considered, has allowed to evaluate changes caused on the intestinal microbiota in presence of helminths, which will potentially provide relevant and applicable information to understand infection in humans (Nguyen et al., 2015; Park and Im, 2020). Such approach lends itself to better monitor and control the infectious process and the many variables that influence the composition of the intestinal microbiota of the host (Lewis et al., 2006; Klementowicz et al., 2012; Houlden et al., 2015; Kreisinger et al., 2015; Martín et al., 2016; Douglas, 2019; Wang et al., 2019; Rosa et al., 2021).

One of the first approaches attempting to identify potential relationships between helminth infections and the host derived factors dates back to 1967 by Wescott *et al*, who using a comparative murine model including both germ-free versus conventional mice, showed that infection by the roundworm *Heligmosomoides polygyrus*, (previously *Nematospiroides dubius*) evolved and persisted in conventional mice, thus suggesting, that factors related to the microbiota were directly related to the development and pathophysiology of this parasite (Wescott, 1968). Since then, numerous studies have been carried out with a variety of other helminths to elucidate how this parasite-mammalian microbiota-host relationship influences host immunity and disease course (Glendinning et al., 2014; Reynolds et al., 2015; Gause and Maizels, 2016; Leung et al., 2018; Midha et al., 2021).

## Host-Helminth-Gut Microbiota Interactions: *Trichuris*

Several descriptive animal model studies have been implemented in an effort to characterize helminth and host microbiota interactions and to determine the effects of infection on host microbiota composition. One of these studies, which implemented a murine model aiming to evaluate helminth-microbiota interactions in *Trichuris muris* infection, offered the view that infection, persistence, and chronicity with this parasite were associated with changes in the composition of the host microbiota. Moreover, further evidence from *Escherichia coli* cultures revealed that the presence of this bacterium allowed a much quicker and more efficient hatching of *T. muris* eggs, hence promoting the establishment of infection (Hayes et al., 2010). Subsequently, analyses carried out in a swine animal model, reinforced the idea that the presence of *Trichuris*, in this specific case of *Trichuris suis*, was also associated with changes related to the intestinal host microbiota. Here the researchers identified that changes occurred mainly in the proximal colon exhibiting an increased presence of the *Mucispirillum*, *Succinivibrio* and *Ruminococcus* genera. Likewise, alterations in fatty acid and carbohydrate metabolism were also evident suggesting a possible link to changes at the microbiota level and providing evidence on the potential effects of parasite influence on the enteric microbiota (Li et al., 2012; Wu et al., 2012).

In 2015, Houlden et al. examined how chronic *Trichuris muris* infection could potentially induce changes in microbiota of C57BL/6 mice (Houlden et al., 2015). Here, the authors identified significant microbiota changes occurring between 14- and 28-days post-infection and which persisted up to 91 days of follow up. Such changes were reflected in the relative abundance of certain taxa, alpha and beta diversity of infected mice and predominantly in *Prevotella* and *Parabacteroides* populations. Most importantly, these changes in the microbiota correlated with changes at the metabolome level, which in agreement with previous studies, revealed a reduction in carbohydrate metabolism and a concomitant increase in certain amino acids, a scenario known to promote a permissive environment for parasite development. However, one of the most interesting findings of this study was the fact that, upon elimination of *Trichuris* infection, a complete recovery of the host intestinal microbiota followed with a simultaneous regaining of the Th1 cell population, which was initially skewed to a Th2 profile during the course of infection. This finding is of utmost importance from the helminth-host interface perspective, since it suggests that *Trichuris* infection is capable of evoking immune regulatory mechanisms and adaptations to the host enteric niche as well as modifying the intestinal microbiota composition favoring parasite persistence, with subsequent restoration of homeostasis after infection (Houlden et al., 2015).

Unquestionably, results obtained from animal models offer very interesting observations that fuel knowledge on parasite-microbiota-host interactions while providing fertile ground for further investigations in the field. However, and despite that animal models may accurately replicate *Trichuris* infection as seen in humans, to date, no experimental comparisons have been trialed in order to validate consistent microbiota changes between animal and human models. In a more recent study, Rosa et al. were able to identify changes in the relative abundances of certain bacterial taxa in the context of *Trichuris* infection, which were consistent in both mouse and human microbiota (Rosa et al., 2021). This study is of particular significance as it allowed to experimentally validate the use of *Trichuris muris* infection in mice as a model to assess *Trichuris*-induced modifications of the human gut microbiome (Rosa et al., 2021). Eleven specific bacterial genera of interest were identified, mainly *Escherichia* and *Blautia*; hence, reinforcing the hypotheses of synergy among *Trichuris* and certain bacterial genera to promote infection by inducing changes in the composition and structure of the intestinal host microbiota (Rosa et al., 2021). At the same time, this study served to validate how animal models offer an important methodological advantage in picturing the many aspects related to host-helminth-gut Microbiota interactions, and its application specifically for the case of *Trichuris* sp. Likewise, a study conducted in Tanzania that sought to determine interactions between parasitic helminths and intestinal microbiota in wild tropical primates from Tanzanian habitats, showed that *Trichuris* sp. was associated with a higher bacterial richness and diversity. These findings could have a relevant ecological impact on different primate populations, highlighting again the importance of interactions between helminths and host microbiota (Barelli et al., 2021).

One of the most conclusive studies examining the *Trichuris*-host microbiota regulation hypothesis was recently published by Schachter et al., in 2020 (Schachter et al., 2020). In their study, using a Swiss Webster mouse model, the authors confirmed the occurrence of changes in the intestinal microbiota and the important immunomodulatory effects induced by *Trichuris muris*, which are essential for the establishment of infection, progression of the parasite’s life cycle and persistence, as well as to promote chronic infections (Schachter et al., 2020). Furthermore, a subset of dexamethasone-induced tolerant mice mirroring a stage of chronic infection, was followed in order to record changes in the intestinal microenvironment. Here, *T. muris* infected mice developed weight loss, anemia, imbalance of the intestinal microbiota with a significant increase of *Escherichia coli* and *Bacteroides* bacteria, enhanced tissue damage at the intestinal level and changes in the Th1/Th2 immune profile (Schachter et al., 2020). These findings reinforce the fact that certain bacterial taxa are essential for an adequate development of the helminth, while concurrently promoting a permissive intestinal environment for it to thrive. It also emphasizes the need to better understand these interactions in order to elucidate those mechanisms linked to clinical progression of the infection and ultimately to evaluate and implement novel control and treatment strategies (Gazzinelli-Guimaraes et al., 2020; Schachter et al., 2020).

Many of the results found in previously described animal models have been consistent with those evidenced in studies with human populations. Lee et al., 2014, conducted an analysis in an indigenous human population in Malaysia, where they observed an increase in the diversity and abundance of the intestinal microbiota in *Trichuris*-infected subjects, with a preponderance of members of the Paraprevotellaceae family, raising evidence that helminths may have an impact in modulating diversity, bacterial community structure and function of the intestinal microbiota of human hosts, being consistent with findings in mice and primates (Lee et al., 2014; Houlden et al., 2015; Barelli et al., 2021). Interestingly, a previous study carried by Lee et al. hypothesized that the presence of *Trichuris trichiura* was not associated with alterations in the host microbiota, as opposed to *Ascaris lumbricoides* (Cooper et al., 2013). This study conducted in Ecuadorian children failed to demonstrate any changes in the composition and structure of the intestinal microbiota, highlighting the variation across human studies and demonstrating that discrepancies may be related to other aspects inherent to the populations evaluated such as age, diet, lifestyle, etc.

A recent systematic review evaluated the impact on the microbiota caused by helminths. This study found that in the case of *Trichuris* infection there was a positive association with *Treponema* sp., *Anaerovibrio* sp., *Rikenellaceae* and multiple species of Prevotellaceae. The authors propose that changes in the Firmicutes/Bacteroidetes (Bacillota/Bacteroidota) ratio, which has been shown to be a biologically relevant ratio, are strongly related to the presence of helminths, causing a shift from Bacteroidetes (Bacteroidota) to Firmicutes (Bacillota) and Clostridia, suggesting that helminth-induced enterotyping may have implications for host health (Whitman et al., 2018; Kupritz et al., 2021). Likewise, in general, helminth infections are associated with greater richness and abundance and this difference is even more tailored when co-infections between different helminths occur, thus highlighting that the interaction of helminths with the host has a great relevance in the changes that can be potentially associated with health and disease (Kupritz et al., 2021).

**Figure 1:**
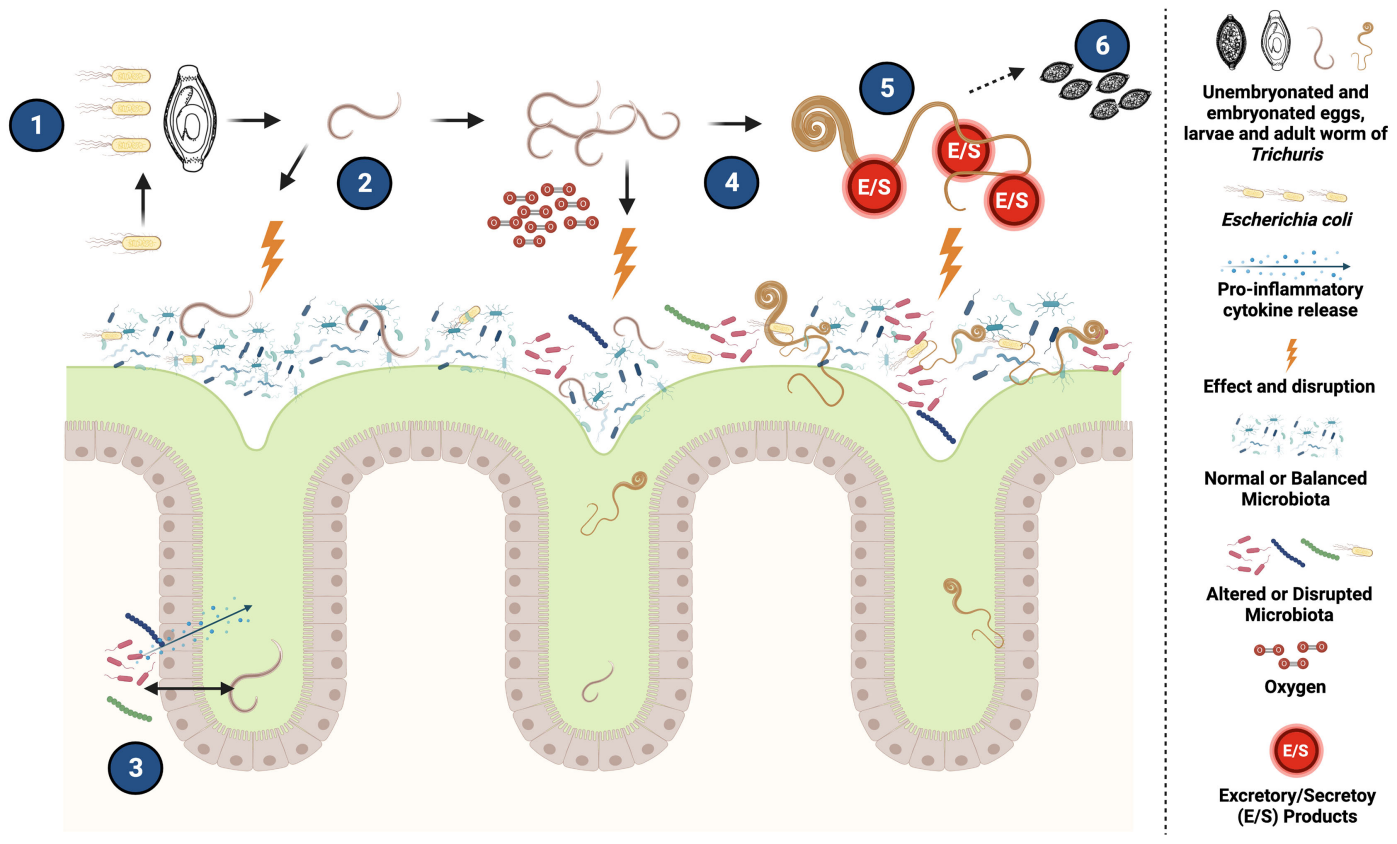
Potential mechanisms of interaction between *Trichuris* and the host intestinal microbiota. (1) After ingestion of the infective stage of *Trichuris*, it has been demonstrated that certain bacteria, such as *Escherichia coli*, can improve the hatching of the eggs, contributing to the establishment of the infection and the development of the parasite. (2) The presence of eggs and released larval stages could cause changes by direct interaction with the host microbiota and by the inflammatory response generated by the parasite (3) Likewise, damage to the intestinal epithelium caused by *Trichuris* can cause translocation of bacteria into the intestinal lumen that may trigger localized inflammatory responses that could potentially affect certain commensal bacterial community of the microbiota influencing the composition and structures of the host bacterial communities. (4) The presence of the parasite in the intestine, together with the changes previously caused in the microbiota, generate an increase in oxygen availability and cause a decrease in strict anaerobic bacteria essential for intestinal health (such as *Faecalibacterium*) and facilitate the increase of facultative aerobes such as some Enterobacteriaceae that in turn contribute to the development of the infection. (5) Adult forms of *Trichuris* can generate Excretory/Secretory (E/S) products that, in addition to having an immunomodulatory effect, can have an impact on certain bacterial groups of the intestinal microbiota, modifying its structure and composition. (6) The adult forms produce eggs that that will be eliminated in the feces. Created with BioRender.com.

Despite the number of studies carried out so far, the specific mechanisms involved in the *Trichuri*s- enteric microbiota interaction remain largely unexplored. Evidence from different studies suggests that *Trichuris* infection is associated with changes in the host microbiota that can potentially lead to increased susceptibility to infection and persistence (Houlden et al., 2015; Vejzagić et al., 2015; Gazzinelli-Guimaraes et al., 2020; Rubel et al., 2020; Rosa et al., 2021), however, further studies are needed to confirm this hypothesis and to understand the mechanisms involved (**Figure 1**). Remarkably, the predominance of certain bacterial taxa has been associated with a better and more efficient hatching of *Trichuris* eggs, as demonstrated in the case of *Escherichia coli* (Wescott, 1968; Rosa et al., 2021). Survival and persistence of the parasite in the intestinal niche is in part driven by modulation of the immune response as a consequence of changes induced at the microbiota level. Likewise, changes related with bacterial composition of the microbiota, particularly the Bacteroidetes (Bacteroidota) (Whitman et al., 2018), including the genera *Prevotella*, *Parabacteroides*, *Paraprevotella*, among others, press for directing further studies in this area since the mechanisms involved in the relationship with specific taxa are yet unclear.

In addition to that concerning to the composition and structure of the microbiota, defining the changes related to the metabolome is perhaps another prominent field of study. Available evidence suggests that in presence of chronic *Trichuris* infection, there is an increased concentration in fresh stool samples of amino acids such as phenylalanine, serine, threonine, leucine, ornithine and glycine, with a decrease in the abundance of vitamin D2/D3 derivatives, fatty acids such as butyrate and glycerophospholipid metabolites related with the mice control group (Houlden et al., 2015). These changes appear to be associated with distinct metabolic profiles in *Trichuris* infection while reflecting how this parasite in context of an altered microbiota may utilize different host resources to enable infection and survival. In this sense, *Trichuris* infection seems to trigger a number of helminth-induced changes in the intestine that lead to a less favorable environment for strict anaerobic bacteria, which are responsible for butyrate production and maintenance of intestinal homeostasis, thus, altering bacterial composition and structure to promote its survival. An example of this is the increase in abundance of facultative anaerobes such as *Escherichia coli*, which has shown to be a determinant factor in the hatching of *Trichuris* eggs.

It is also worth mentioning that beyond direct parasite-bacteria interactions, indirect interactions mediated by parasite derived molecules, can also play a role in the immunomodulatory effects leading to modifications of the structure and composition of the host microbiota. For example, it has been described that *Trichuris* exhibits the ability to generate excretion/secretion (E/S) products containing different molecules to facilitate survival and persistence of the helminth. A recent study demonstrated that amongst these E/S products, the p43 protein shows the ability to inhibit IL-13, which is an essential effector cytokine necessary for parasite clearance (Bancroft et al., 2019). Thus p43 exerts its immunomodulatory effects by allowing not only the survival of *Trichuris*, but also progression to chronic stages of infection (Bancroft et al., 2019). These types’ of helminth-derived immunomodulatory molecules may also interact indirectly with the host microbiota leading to possible changes in diversity and abundance and at the same time favoring parasite invasion with its consequent pathophysiological chain of events (Bancroft et al., 2019; Schachter et al., 2020; Lawson et al., 2021; Midha et al., 2021).

It is evident from the different studies carried out on *Trichuris*-microbiota-host interactions that this helminth inflicts significant effects on the intestinal microbiota by means of different mechanisms (**Figure 1**). These changes affecting the composition and structure of the host microbiota may lead to a complex regulatory cascade in the intestinal niche which favor helminth infection, survival and persistence, ultimately impacting the course of infection. More studies focusing on how this parasite affects the composition of the enteric microbiota and its impact on host biology are required.

## Host-Helminth-Gut Microbiota Interactions: *Ascaris*

Studies on *Ascaris*-Microbiota-Host interactions have also been carried out from descriptive designs, both in human populations and animal-based models. A distinct feature for *Ascaris* is that, during this nematode’s life cycle, short after ingestion of eggs, the larvae (L3) have the ability to invade mucosal tissue of the gastrointestinal tract and subsequently migrate towards hepatic and pulmonary tissues (Sultan Khuroo, 1996; Nogueira et al., 2016; Midha et al., 2021; Wu et al., 2021). In the lung, infection triggers and inflammatory response characterized by eosinophil infiltration (Wu et al., 2021). The migratory nature of certain parasite stages has also been linked to possible modifications on the composition and structure of the intestinal microbiota. An evidence of this is the fact that translocation of helminth associated bacteria through the intestine barriers can be traced short after, to the lung mucosa. In fact, bacterial translocation can trigger localized inflammatory responses that can potentially affect certain commensal bacterial community of the microbiota (Adedeji et al., 1989; Hübner et al., 2013; Midha et al., 2021). In a study by Cooper et al. in 2013, conducted in Ecuadorian populations the authors present evidence that *Ascaris lumbricoides* in contrast to *Trichuris* does impact intestinal microbiota of the host (Cooper et al., 2013). The authors also suggest that a single infection with *Ascaris lumbricoide*s or co-infection with other helminths can lead to alteration of the microbiota profile by exhibiting lower relative abundances of certain taxa, predominantly Clostridia class and higher relative abundances of bacteria belonging to the genus *Streptococcus*. Similarly, they propose that the presence of this helminth is linked to a lower diversity in the host microbiota (Cooper et al., 2013). These differences between *Ascaris* and *Trichuris*, may be influenced by several factors such as studying samples from a rural population or evaluating the presence of *Ascaris* in context of mixed infections with *Trichuris* and not as a single infection. The results described above were subsequently addressed in a swine model infected with *Ascaris suum*, which sought to find potential effects that could be extrapolated to studies in human infection (Wang et al., 2019). Here, the authors demonstrated that infection with this helminth significantly reduced the alpha diversity of the intestinal microbiota of pigs and that this effect was independent of the parasite load. Also, they were able to determine a differential relative abundance associated with a profile of at least 49 bacterial genera, including *Prevotella* and *Faecalibacterium* (Wang et al., 2019). Additionally, changes in the metabolic profile of stool samples from helminth-infected pigs was observed, noticing a decrease in carbohydrate and amino acid metabolism, in accordance with those studies conducted in *Trichuris* infections (Houlden et al., 2015). As for the case *Trichuris*, discordant results stand out amongst different studies. For example, while the study by (Wang et al., 2019) found a reduced alpha diversity in the intestinal microbiota of pigs infected with *Ascaris suum*, a previous study by Williams et al. in 2017 found that *Ascaris* modified the host’s intestinal microbiota with an increased alpha diversity but a concomitant reduction in the relative abundance of the genera *Lactobacillus*, *Ruminococcus* and *Catenibacterium* (Williams et al., 2017). These observations underscore the importance of using appropriate epidemiological designs and animal models inclusive of a greater number of infection-associated variables that can be controlled and impact on host intestinal microbiota changes, as well as the need to establish experimental protocols and reproducible bioinformatic analyses to allow validation of the results obtained in these research studies.

A recent study carried out in a Thai population, which assessed the presence of helminths, including roundworms (*Ascaris lumbricoides*), whipworms (*Trichuris trichiura*) and hookworms (*Ancylostoma* spp. and *Necator americanus*), allowed to identify changes in the microbiota of infected individuals (Stracke et al., 2021). Although no differences were apparent in reference to alpha diversity at the bacterial level, marked changes impacting beta diversity were noticeable. Likewise, modifications were found for certain bacterial taxa, where a greater abundance of *Akkermansia*, *Prevotella*, *Bacteroides coprophilus* and *Ruminococcus* and a decrease in *Bifidobacterium* was observed. The foregoing facts signal the influence that the presence of helminths such as *Ascaris* and *Trichuris* have on the composition and structure of the host’s intestinal microbiota and how this may translate into the pathophysiology of the disease (Stracke et al., 2021). As previously discussed, a key aspect of this study is that it emphasizes that the uncertainty associated with the evaluation on the impact of helminth infection on the intestinal microbiome is probably not only related to the complexity of the interaction but also linked to the technical aspects of the experimental design and analytical approach, weighing the complex limitations involved in the process such as size sampling, collection, preservation and transport of samples, among others (Stracke et al., 2021).

One main aspect that needs to be considered in *Ascaris*-microbiota-host interactions is the fact that infection by this parasite can increase susceptibility to infection by other pathogens, mainly bacterial. In swine models, it has been shown that infection by *Ascaris suum* (Wang et al., 2019) and *Trichuris suis* (Li et al., 2012) are related to a higher relative abundance of *Campylobacter jejuni*, suggesting that these helminths could potentially increase the risk for secondary infections to this bacteria, an aspect of great relevance from a clinical and public health perspective.

As for the case of *Trichuris*, *Ascaris* derived excretory-secretory products may also promote an immunomodulatory response linked to indirect interactions with the intestinal microbiota of the host. E/S products, extracellular vesicles and other metabolites have been identified which may interact in different ways with the host microbiota and may play a key role in parasite biology and pathophysiology of infection (Hansen et al., 2019). In these lines, studies have confirmed that *Ascaris suum* has the ability to induce production of ASABF (*Ascaris suum* antibacterial factor) type antimicrobial peptides (Pillai et al., 2003; Midha et al., 2018) and microbial peptides of the cecropin family (P1 to P4) in the host. These substances have shown to exhibit bactericidal effect against a wide range of microbes, such as gram-positive (*Staphylococcus aureus, Bacillus subtilis* and *Micrococcus luteus*) and gram-negative (*Pseudomonas aeruginosa*, *Salmonella typhimurium*, *Serratia marcescens* and *Escherichia coli*) bacteria (Pillai et al., 2005). This highlights the ability of *Ascaris* to produce a number of nematode-derived metabolites which can exert important modulating effects on the host intestinal microbiota, thus constituting one of the most trending aspects in the field of helminth biology.

**Figure 2:**
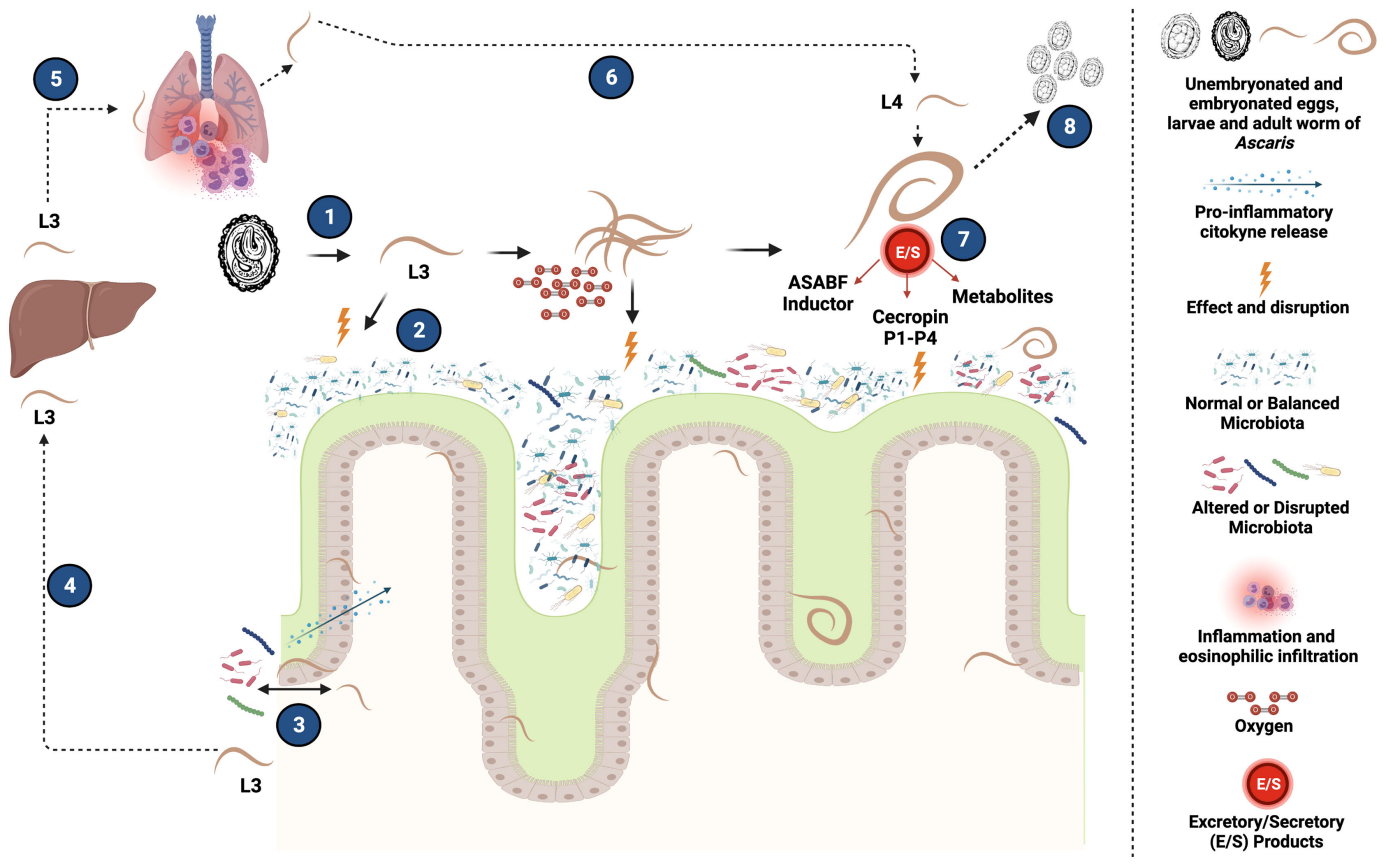
Potential mechanisms of interaction between *Ascaris* and the host intestinal microbiota. (1) After ingestion of the infecting stage of *Ascaris*, L3 larvae are released from embryonated eggs into the host intestine. (2) These larvae can have an effect by direct interactions on the intestinal microbiota and thus propitiate an increase in oxygen availability that promote the increase of facultative aerobic bacteria and the decrease of strict anaerobic bacteria necessary for an adequate intestinal health, generating a disruption of the host microbiota. (3) Additionally, within the life cycle of *Ascaris*, damage to the intestinal epithelium caused to continue its migration produces the translocation of bacteria into the intestinal lumen, that may trigger localized inflammatory responses that could potentially affect certain commensal bacterial community of the microbiota influencing the composition and structures of the host bacterial communities (4) Migration of *Ascaris* L3 larvae to the liver and subsequently (5) from the liver to the lungs, allowing *Ascaris* to complete later phases of its life cycle, generating, among other things, inflammatory processes at the level of the pulmonary tissue characterized by a high infiltration of eosinophils and clinical symptoms such as those evidenced in Loeffler’s Syndrome. (6) Larvae released from the lung can migrate back to the intestine and transform into L4, for later completing their development into adult forms. (7) These adult forms have the property of generating Excretory/Secretory (E/S) products and other metabolites with great immunomodulatory capacity, and also with antimicrobial function, such as ASABF inducers and Cecropin P1 to P4 proteins, that can modify the structure and composition of the host intestinal microbiota. (8) Finally, in an environment with now beneficial conditions for *Ascaris*, the adult forms have the ability to produce eggs that will be eliminated in the feces to continue its life cycle and infection process. Created with BioRender.com.

The biological characteristics of *Ascaris* infection, which includes multifaceted aspects such as extra-intestinal migration, modulation of the host immune response, and in context of this review, the changes it can inflict on the host’s microbiota, altogether blends into a complex network of mechanisms that subvert the helminth-mammalian host relationship allowing the parasite to establish infection and promote its survival in the host (**Figure 2**). Studies in both humans and animal models have defined how *Ascaris* displays its ability to generate changes in both the structure and composition of the host microbiota *via* direct interaction or through production of metabolites and different E/S products, such as antimicrobial peptides, that can further restructure predominant bacterial community. This has a particularly detrimental effect on strict anaerobic bacteria which are essential for proper intestinal functioning such as the case of *Faecalibacterium* (Wang et al., 2019) and *Ruminococcus* (Williams et al., 2017). Alongside, a concomitant increase of facultative anaerobes such as *Streptococcus* (Cooper et al., 2013), which coupled with the immunomodulatory effects of this helminth, allows it to provide a suitable metabolic environment to ensure its survival and reproduction. Thus, it is evident that alterations and subsequent effects on the host microbiota have a fundamental role in the biology and pathophysiology of the parasite paving the way in search for therapeutic alternatives based on the reestablishment of normal host microbiota. Likewise, it is important to highlight that the immunomodulatory capacity of this helminth and its impact on the host microbiota can also promote a greater susceptibility to suffer reinfections or co-infections by other bacterial pathogens. Therefore, enhancing our knowledge on the mechanisms in play during early phases of infection are crucial to succeed in preventing adult worm establishment and the deleterious effects of helminth and host-derived factors during their interaction in the enteric milieu.

## Conclusions and Future Perspectives

The study of parasite-host-microbiota interactions is part of a field of great interest as it can provide new insights and provide key aspects in the biology, dynamics and pathophysiology of infection. The high prevalence of helminths in developing countries, high rates of reinfection and resistance to anthelmintics and current unavailability of vaccines against these parasites, stress the need to focus research into elucidating the mechanisms involved in these interactions and thus identify essential aspects pertaining the design of new strategies to control the spread of these helminths as well as novel therapeutic alternatives. In this review, we have included the nematodes *Trichuris* and *Ascaris* since they exhibit the highest prevalence and level of endemicity in low and middle-income countries, as well as to their notorious impact on animal health and the swine industry (Sayasone et al., 2011; Walker et al., 2013; Ojha et al., 2014; Pullan et al., 2014; Dunn et al., 2016; Cortés et al., 2019; Hernández et al., 2019; Sobotková et al., 2019; Else et al., 2020; Gordon et al., 2020).

Different studies have shown that both *Trichuris* and *Ascaris* interact in important ways over the host microbiota through several direct or indirect mechanisms. This interaction provokes profound changes in the composition and structure of the microbiota allowing the parasite to establish, thrive and progress to chronic stages of infection. These changes promote a favorable metabolic, microbial and immunological environment for these parasites, which may lead to a greater susceptibility to acquire superinfections by other pathogenic bacteria such as *Campylobacter*, as has been demonstrated in swine models (Glendinning et al., 2014; Kreisinger et al., 2015; Loke and Lim, 2015; Giacomin et al., 2016; Midha et al., 2017, []2021; Williams et al., 2017; Leung et al., 2018; Lawson et al., 2021; Rosa et al., 2021). In light of these observations, it is essential to continue to conduct studies that allow us assess the potential benefits of probiotics as a novel and effective therapeutic alternative against helminth infections.

In addition, it is necessary to carry out studies, which examine not only bacterial community of the intestinal microbiota, but also eukaryotic community that are also an important component of this microenvironment. Despite advances in sequencing technologies and bioinformatics tools, most studies involving the intestinal microbiota are still limited to bacterial communities and very little is known about how eukaryotes and virus influence and determine the composition, structure and functioning of the host gut microbiota and its interaction with parasites. To date, different protozoa and fungi have been linked with potential changes in the host gut microbiota (Mason et al., 2012; Iebba et al., 2016; Nagel et al., 2016; Stensvold and van der Giezen, 2018; Partida-Rodriguez et al., 2019; Castañeda et al., 2020). Also, it is important to consider that helminth infections can coincide with other protozoa defining intestinal polyparasitism events (Sayasone et al., 2011; Salcedo-Cifuentes et al., 2012; Cimino et al., 2015; Donohue et al., 2019; Geus et al., 2019; Gordon et al., 2020). For example, studies have demonstrated how the presence of the protozoan parasite *Blastocystis* can induce changes in the diversity and abundance of the bacterial microbiota, favoring an increase in taxa such as *Faecalibacterium* while decreasing others such as *Bacteroides*. This is similar to the well-known beneficial relationship of this protozoan with the host microbiota by generating an anti-inflammatory environment (Audebert et al., 2016; Iebba et al., 2016; Castañeda et al., 2020). Another case of mutualistic interaction has been described for the protozoan *Tritrichomonas musculis*. This eukaryote shows the ability to activate the host epithelial inflammasome inducing the production of IL 8, which in turn promotes activation of the Th1/Th17 axis, eliciting protection against bacterial infections of the mucous membranes, and ultimately preventing the potential increased risk of suffering inflammatory diseases (Chudnovskiy et al., 2016). Both animal and *in vitro* studies have equally demonstrated that protozoa such as *Giardia* exhibit the capacity to promote alterations in host microbiota, leading to an increased predisposition to develop inflammatory and metabolic diseases (Iebba et al., 2016; Barash et al., 2017; Toro-Londono et al., 2019). As well as eukaryotes interact with the intestinal microbiota, viruses can also influence changes in its composition. The main proportion of the human virome is constituted by bacteriophages which play a key role in the structuring and flow of information amongst bacterial and microbiota components, thus, regulating in a critical way co-evolution events shared between both of these components (Moreno-Gallego et al., 2019; Mukhopadhya et al., 2019; Hufsky et al., 2020). Similarly, it has been hypothesized that the enteric virome may be intimately linked to the etiopathogenesis of gastrointestinal diseases. Both bacteriophages and eukaryote derived viruses may infect host cells and undermine the host’s own bacterial microbiota under certain conditions, triggering immunological responses which induce changes in the structure and composition of the microbiota. Therefore, it is essential to recognize not only the role of bacteria, but also eukaryotes and viruses and their interaction across the host-helminth-gut Microbiota interface, as this may reveal key information about the mechanisms associated with these interactions.

Furthermore, it is imperative to look into the helminth’s own microbiota, since it not only defines the transient microbiota specific to each stage of the life cycle and its relationship with the host, but also because it can reveal mutualistic associations between certain bacterial groups and the parasite which may prove essential to its biology and ability to infect and disseminate (Jenkins et al., 2019; Formenti et al., 2020). For example, *Wolbachia* bacteria have been found in nematodes of the family Onchocercidae, a family of filarial nematodes, which includes clinically relevant species known to cause lymphatic filariasis and onchocerciasis and which are of vital importance to the parasite since they provide essential metabolites to the filariae as part of a symbiotic relationship (Bouchery et al., 2013). Bacteria of the genus *Neorickettsia* have also been identified in *Fasciola* trematodes through genomic analysis, thus, suggesting a potential mutualistic relationship between these organisms (McNulty et al., 2017). Very little is known about the establishment, structure and function of the microbiota residing in these nematode parasites and therefore, there is a critical need to evaluate these characteristics in an attempt to better decipher the interactions between the parasite and the host microbiota.

**Figure 3:**
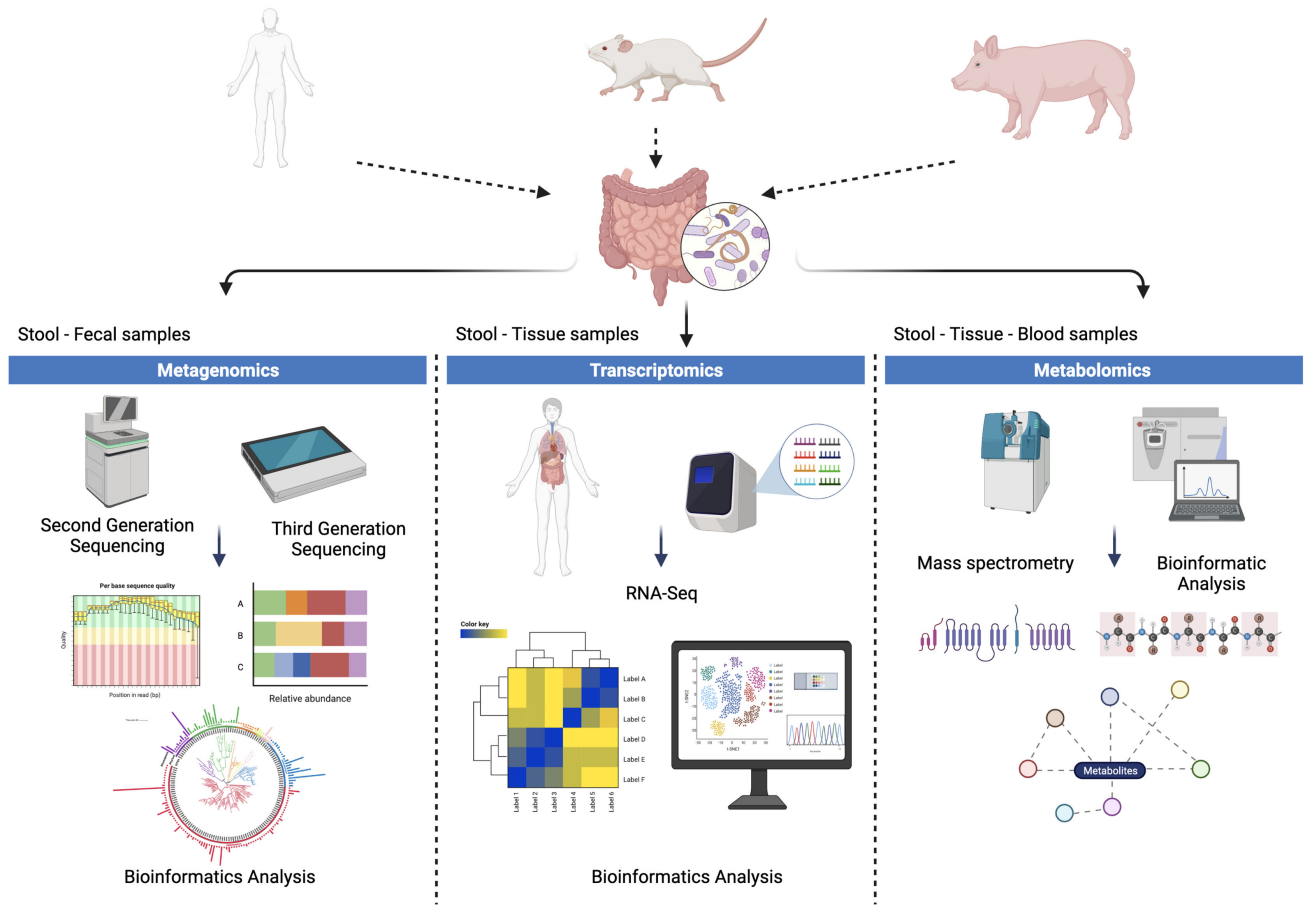
Methodological approaches for the study of parasite-microbiota-host interactions. Studies in humans and in animal models, such as murine and porcine models, facilitate the evaluation of helminth-host microbiota interactions from different approaches such as: (i) metagenomic approaches by means of second and third generation sequencing, (ii) approaches based on transcriptomics and spatial transcriptomics and (iii) metabolomic approaches based on techniques such as mass spectrometry.

There are still many gaps in knowledge on how interactions between the parasite and host microbiota may confer biological advantages that facilitate infection and dissemination, and how these relate to the pathophysiology and clinical manifestations of infection and disease. In order to better understand the complex mechanisms involved in these parasite-microbiota-host interfaces, both in humans and animal models, it becomes critical to design multifaceted investigative approaches (**Figure 3**). Metagenomic analyses using second- and third-generation sequencing technologies can yield key information related to the structure and composition of the microbiota, including prokaryotic, eukaryotic and viral profiles. Such approach can also facilitate the prediction of genes and metabolic pathways that may be differentially expressed in helminth-infected individuals. Transcriptomic and spatial transcriptomic (that enables high-resolution assessment of spatial gene expression across tissue sections) studies will allow evaluation on the differential behavior of gene expression in the enteric niche (or respiratory tract as for the case of *Ascaris*), provide information related to tissue tropism, location and migration patterns of the parasite. Finally, metabolomics mass spectrometry-based studies, for example, from feces and blood samples, could potentially contribute to our knowledge on chemical and metabolic changes related to helminth infections, and how these correlate with alterations in the composition and structure of microbial communities. It is important to emphasize at this point that it is indispensable to carry out these studies under these methodological approaches in such a way that their reproducibility can be assured. It is essential to control, mainly in descriptive studies with humans, the multiple variables that can influence the composition of the microbiota and that can produce biases in the results if they are not appropriately controlled (age, gender, rural or urban population, diet, breastfeeding, route of birth, among others) (Sadowsky et al., 2017; Rinninella et al., 2019). The use of animal models has favored standardization of processes allowing adequate control of most of the variables involved in microbiota analysis. However, it is important to emphasize that in order to translate results derived from mouse studies into a human context, it is necessary to take into account similarities and differences between the gut microbiota of both mice and humans. The comparative physiology of the intestinal tract, the effect of dietary patterns, and differences in genetic backgrounds must also be considered. It is therefore essential to continue to develop and implement humanized gnotobiotic mouse models that allow more concrete and efficient extrapolation of knowledge to the human context (Nguyen et al., 2015; Hugenholtz and de Vos, 2018; Park and Im, 2020). Additionally, the ever-increasing availability of bioinformatics tools should be carefully examined to define their relevance according to the protocols and types of methodologies and analyses, allowing for greater comparability of studies and results. The information obtained from approaches such as those mentioned above are essential for the generation, evaluation and implementation of possible therapeutic alternatives and potential improvements in control mechanisms particularly in areas with high prevalence of these parasites and where reinfections are frequent despite anthelmintic treatment

It has been identified that the microbiome can offer important advantages in the prediction and diagnosis based on the characterization of certain markers, both associated with health and with different types of diseases such as gastric cancer, and it has also been sought to extrapolate this advantage to the prediction of aspects related to parasitic infections in order to establish relationships and even to predict factors such as the parasitic load and the prognosis of the infection (Gupta et al., 2020; Zhang et al., 2021). In this context, Rubel et al. in 2021, in Cameroonian populations, from metataxonomic and metagenomic analyses and through the implementation of machine learning strategies such as Random Forest, managed to identify those members of the order Bacteroidales, which are common members of the intestinal mucosal surfaces, and are known as an important predictive taxon of infections caused by soil-transmitted helminths and conversely, that the presence of *Ruminococcus bromii*, may be a marker of protection against helminth infection (Rubel et al., 2020). This highlights the importance of focusing studies to identify markers that can facilitate the prediction, prognosis and diagnosis of parasitic infections caused by helminths.

Further studies are needed to validate this hypothesis and to establish an appropriate management framework for the potentially applicability and safe use of these alternatives. Thanks to advances in sequencing methodologies and bioinformatics tools and analyses, the development of these studies in humans and animal models will facilitate the integration of essential knowledge for the understanding of parasite-host interactions.

## Author Contributions

JR and SC conceived the objective of the review. SC, AP-M, and JR wrote and revised the final version of the manuscript. All authors contributed to the article and approved the submitted version.

## Funding

SC is funded by Programa de Becas de Excelencia Doctoral del Bicentenario, definido en el artículo 45 de la Ley 1942 de 2018 from the Ministerio de Ciencia, Tecnología e Innovación de Colombia.

## Conflict of Interest

The authors declare that the research was conducted in the absence of any commercial or financial relationships that could be construed as a potential conflict of interest.

## Publisher’s Note

All claims expressed in this article are solely those of the authors and do not necessarily represent those of their affiliated organizations, or those of the publisher, the editors and the reviewers. Any product that may be evaluated in this article, or claim that may be made by its manufacturer, is not guaranteed or endorsed by the publisher.
